# Assessing research misconduct in Iran: a perspective from Iranian medical faculty members

**DOI:** 10.1186/s12910-021-00642-2

**Published:** 2021-06-21

**Authors:** Erfan Shamsoddin, Zahra Torkashvand-Khah, Ahmad Sofi-Mahmudi, Leila Janani, Payam Kabiri, Ehsan Shamsi-Gooshki, Bita Mesgarpour

**Affiliations:** 1 National Institute for Medical Research Development (NIMAD), West Fatemi St., Tehran, Tehran, 1419693111 Iran; 2 Department of Biostatistics, School of Public Health, Iran University of Medical Sciences, Tehran, Iran; 3 Department of Biostatistics and Epidemiology, School of Public Health, Tehran University of Medical Sciences, Tehran, Iran; 4 Department of Medical Ethics, Faculty of Medicine/Medical Ethics and History of Medicine Research Center, Tehran University of Medical Sciences, Tehran, Iran

**Keywords:** Scientific misconduct, Surveys and questionnaires, Biomedical research, Iran

## Abstract

**Background:**

Research misconduct is a global concern in biomedical science. There are no comprehensive data regarding the perception and situation of scientific misconduct among the Iranian medical faculty members. We conducted a nationwide survey to assess the research misconduct among the medical faculty members in Iran.

**Methods:**

We used the Persian version of the research misconduct questionnaire (PRMQ) on the Google Forms platform. We sent the survey link to a systematic random sample of medical faculty members in Iran (*N* = 4986). Descriptive analyses were performed on the individual items of the PRMQ, with frequencies and percentages for categorical and Likert-type response items, and means and standard deviation (S.D.) for continuous variables. Chi-square analysis was conducted to test hypotheses examining differences in the frequency of responses related to factors influencing misconduct. We also defined four tenure categories (TC) based on the working years of the participants as tenured faculty members. All the analyses were performed using R 3.6.0.

**Results:**

The response rate was 13.8% (692 responses). Nearly 70% of the respondents agreed that their publication output would be of higher quality if there were no publication pressure. Approximately three-quarters (*N* =499, 72.1%) of the respondents had been aware of some instances of research misconduct during the previous year according to their understanding of misconduct. Among the participants, 18.5% perceived the effectiveness of their associated organisation’s rules for reducing research misconduct to be high or very high. Pressure for tenure was identified as the item most frequently perceived with a strong behavioural influence on engaging in research misconduct (80.2%).

**Conclusions:**

This study confirms that research misconduct needs to be actively addressed among the medical faculty members. Making policies with a focus on boosting awareness regarding the occasions of scientific misconduct and its management seems to be indispensable in the future in Iran.

**Supplementary Information:**

The online version contains supplementary material available at 10.1186/s12910-021-00642-2.

## Background

Research misconduct has been a growing concern in almost every scientific field, including medical sciences [1]. Two of the most recognised definitions for research misconduct are as follows:

1. The US Department of Health and Human Services Office of Research Integrity defines it as “fabrication, falsification, or plagiarism in proposing, performing, or reviewing research, or in reporting research results” [2];
2. The UK Research Integrity Office’s definition includes “a) fabrication, b) falsification, c) misrepresentation of data and/or interests and/or involvement; d) plagiarism; and e) failures to follow accepted procedures or to exercise due care in carrying out responsibilities for subjects and private information” [3].

While these definitions include the most severe unethical behaviours recognised as misconduct, others include misbehaviours such as conflict of interest and misuse of funds [4–6]. Considering their detrimental effects on individuals’ health status (e.g. patients, etc.) and extensive financial costs levied upon healthcare systems, such wrongdoings have even more salience in medical sciences [7–9]. In Iran, the National Committee for Ethics in Biomedical Research defines research misconduct as “any violation of the requirements, regulations, guidances, guidelines, and codes to protect human participants in research, as approved by the Ministry of Health and Medical Education (MOHME) for designing, executing and reporting results of biomedical research, and abuse of intellectual property pertaining to practical and theoretical research findings of other parties” [10].

Even though many consider research misconduct resulting from inadequate training, personal circumstances, and individual behaviour and attitude, factors in the research environment (like poor supervision and competitive pressures) should not be overlooked [11, 12]. Factors influencing the research misconduct occur in three levels: 1) research policies and strategies determined by policymakers (macro-level), 2) research development programs run by universities and academic organisations (meso-level), and 3) research projects conducted by individuals (micro-level) [13]. Thus, a comprehensive, multilevel approach is needed to reduce the research misconduct and its main contributing causes in research systems [12, 14].

Current estimates of the prevalence of research misconduct differ in distinct countries. A multinational retrospective study on previously retracted studies in 2013 showed that in contrast to the USA, Switzerland, and Germany, scientific misconduct in Iran, India, and Turkey had a higher ratio of publication misconduct to mistrusted data in the retracted studies [15]. Another retrospective study showed that countries with the most rapid growth in scientific publications (e.g. China, Malaysia, and Mexico) simultaneously exhibited the highest retraction rates [16, 17].

As concerns have been growing about the erupted cases of medical research misconduct globally [18], efforts have been exerted to measure the extent of such wrongdoings, whereby counteracting this issue will be more effective. As a part of medical research, Iranian medical researchers have noticeably contributed to publishing articles, albeit showing some cases of research misconduct in the previous years [19, 20]. This indicates a need for more information about measuring scientific misconduct in Iran. A few studies have scrutinised the prevalence of various aspects of research misconduct among Iranian medical researchers. A survey on the dissertation of undergraduate and postgraduate medical students at a medical school in 2015 revealed that 19% of undergraduates and 25% of postgraduates had admitted committing misconduct [21]. Another study in academic members of a medical university in Iran showed that a notable proportion of the respondents had engaged in at least one of the so-called top-ten misbehaviours introduced by the paper [22]. Another survey assessing the prevalence of publication misconduct among Iranian medical corresponding authors reported guest authorship, falsification of study methods, and plagiarism as the three most common wrongdoings in Iran during 2009–2011 [19]. Even though many local studies have addressed scientific misconduct in Iran, there have been no comprehensive data regarding the prevalence of research misconduct among medical faculty members in the whole country. Without having these figures or at least an estimate of them, devising effective policies to manage medical research misconduct (either at macro- or meso-level) in Iran will most probably be in vain. This study aimed to conduct a nationwide survey using the Persian version of the research misconduct questionnaire (PRMQ) among the faculty members of medical universities in Iran to evaluate their perceptions, beliefs, practices, and experiences related to scientific misconduct.

## Methods

### Devising the PRMQ

Two questionnaires were considered as sources to be translated to Persian and get their psychometric characteristics checked [23, 24]. Overall, 63 items were devised in seven subscales as follows: perception of the workplace environment, the prevalence of scientific misconduct, awareness of research misconduct, reporting research misconduct, beliefs about research misconduct, behavioural influences, and publication pressure. The final version of the questionnaire is accessible at https://drive.google.com/file/d/1nLhwovk7qVEL7Ztw-wm0PMIzyvKHRCDF/view?usp=sharing. The validity of the Persian version of the research misconduct questionnaire (PRMQ) was assessed qualitatively (face validity from experts and pilot testing) and quantitatively (using content validity index and content validity ratio). Content validity indices were higher than 0.7 for all the items, thereby considered valid. Additionally, reliability was scrutinised using Cronbach’s alpha coefficient, ranging from 0.61 to 0.87 for all these subscales. The detailed steps of translating, validating, and checking the internal consistency of PRMQ are expounded elsewhere [25].

### Participants

The PRMQ items were uploaded as an online survey on the Google Forms platform. After estimating the sample size, we ordered all the medical faculty members in Iran based on their H-index. From the beginning of this sorted list of faculty member (19,944 individuals), one of four members was randomly selected and included in our sample. This repeated process resulted in a systematic random sample of 4986 medical faculty members in Iran—data gathered from the Iranian Scientometric Information Database (ISID). This online database provides an up-to-date pool of scientometric information about the faculty members affiliated with the MOHME in Iran (isid.research.ac.ir). We sent the survey link twice to all of the Iranian medical scholars from March to November 2019. Based on their performance in education, research, and facilities, these universities are categorised into three types by the Ministry of Health and Education in Iran—type one as the highest-ranked and type three as the lowest-ranked. Several criteria are considered for ranking these universities: contribution to science, annual budget, research infrastructures, and human resource development capacity. Accordingly, type one universities are generally more developed and founded earlier than the other two types. Our participants were affiliated with either type one, two, or three medical universities in Iran.

### Data management and analyses

We gathered all data directly from the Google Forms responses. There was no time limit for putting in the answers or any sign-in time restrictions. Participants were free not to answer some specific questions and to leave the survey at any points. Before the beginning of the first section of PRMQ, consent was sought and given. The answers were exported into Microsoft Excel (2019) data sheets; cleaning and sign-posting were conducted using the same software. Descriptive analyses were performed on the individual items of PRMQ, with frequencies and percentages for categorical and Likert-type response items (starting from one to higher integers, i.e. 2,3,4,…), and means and standard deviation (S.D.) for continuous variables. We conducted a Chi-square analysis to test hypotheses examining differences in frequency of responses. Our hypotheses could be related to factors influencing misconduct and reporting it or factors related to personally experienced publication pressure (based on the type of university, being aware of a research misconduct occasion, or the length of serving as a tenured faculty member for each participant).

Tenure categories were defined as follows: (1) tenure category one (TC1) for those who worked in tenure conditions for less than or equal to five years; (2) tenure category two (TC2) for those who worked in tenure conditions for more than five years and less than or equal to 10 years; (3) tenure category 3 (TC3) who worked in tenure conditions for more than 10 years and less than or equal to 15 years; and (4) tenure category four (TC4) for those who worked in tenure conditions for more than 15 years. An overall cumulative score was defined for the publication pressure subscale as the average sum of scores for all items in all the respondents, which could range between 14 and 70. This study was approved by an institutional ethics committee with the code number IR.ACECR.IBCRC.REC.1397.011 in 2018. All the analyses were performed using R 3.6.0 (R Core Team, 2019), the “Tidyverse” package (v1.3.0, Hadley Wickham, 2019), and the “Questionr” package (v0.7.3, Julien Barnier, 2020).

## Results

### Demographic and work setting features

Of 4986 faculty members, 692 responded (13.8% response rate); 393 (56.7%) were male, and 299 were female. The mean (S.D.) age of participants was 46.0 (8.15) years, with a range of 28–71 years. The mean (S.D.) of the years of working as a tenured scholar and H-index of our respondents were 11.2 (9.24) and 7.1 (6.05), respectively. Nearly half (50.3%) of participants were affiliated with type-1 universities; most of them (64.4%) had a PhD degree, while speciality medical practitioners were the second most prevalent (27.1%). Table 1 depicts the participants’ demographic information and work setting features in more detail.

**Table 1:** Demographic characteristics of the respondents at different university types

Characteristic	Type 1	Type 2	Type 3
Respondents' no.	348 (50.3%)	246 (35.5%)	98 (14.2%)
*Gender*			
Male (%)	184 (26.5%)	140 (20.3%)	69 (9.9%)
Female (%)	163 (23.5%)	107 (15.5%)	29 (4.3%)
Age (mean, SD)	47.2 (7.80)	45.2 (8.27)	43.2 (8.11)
Tenure (mean, SD)	12 (8.84)	10.6 (8.61)	9.8 (7.35)
H-index (median, IQR)	7 (8)	4 (6)	4 (4)
*Degree*			
Master	5	21	30
MD	117	59	14
PhD	225	167	54
Total	347	247	98

No.: Number; SD: standard deviation; IQR: inter-quartile range

Almost one-third (31.6%) of the respondents practised in clinical settings, and 80.4% of them mentioned having served as a chair or vice-chancellor in their research organisations. The average period of working as a researcher was 7.9 (SD: 8.86). Among the involved researchers, 84.9% rated their understanding of rules and procedures related to research misconduct as high or very high.

### Awareness of research misconduct

Responding to the question that how did anyone become aware of any case of research misconduct in the past, each individual could select more than one choice from the following: observed the instance themselves, heard from official channels of their organisation, from other research coordinators, from collaborating investigators, from study monitors, or from institutional review board. The results of this question were overlapping. Nearly three-quarters (*N* =499, 72.1%) of the respondents had been aware of some instances of research misconduct anytime before answering. We found no evidence for a difference in the rate of the awareness of misconduct among various scholars from differing university types or with disparate tenure experience categories (*p* = 0.62 and 0.48, respectively).

### Perceived prevalence of scientific misconduct

**Fig. 1:**
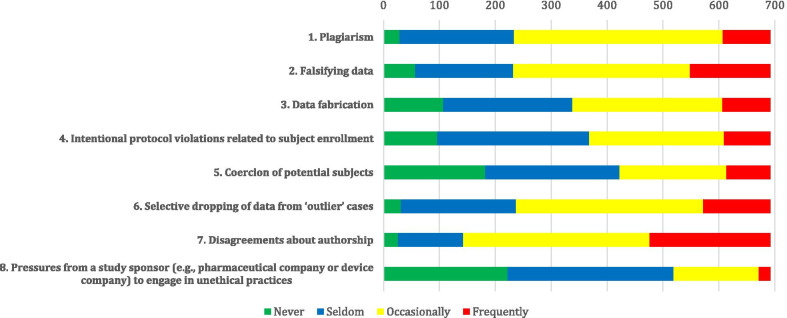
The number of responses to each item in the “prevalence of scientific misconduct” section

The most frequent response in each category of the faculty members’ perception of the prevalence of misconduct in their workplaces was “occasionally.” Disagreement about authorship averagely scored the highest among all the presented misconducts (31.2%). Plagiarism and falsified data were rated to be faced either occasionally or frequently by 66.2% and 66.5% of the participants, respectively. Respondents aware of misconduct were more likely to report higher rates of perceived prevalence of protocol violations related to subject enrolment (df: 3, *p* < 0.001). Figure 1 shows the perceived prevalence of scientific misconduct among our participants.

### Reporting of research misconduct

In answer to the question, what a typical research coordinator would do if they were aware that a principal investigator (PI) or coinvestigator violated research integrity rules, 72.9% of scholars responded that the coordinator would do nothing or opts not to report the occasion. The figure for the same question only regarding a member of the research team or staff member engaging in the wrongdoing was 69.6%. Respondents who rated the effectiveness of their organisational policies in tackling the misconduct as high or very high were more likely to indicate higher chances of the coordinator to report the misconduct (df: 3, *p* < 0.001). However, participants with awareness of misconduct were more likely to indicate that a typical research coordinator would probably do nothing (df:3, *p* < 0.001). Those affiliated with type 3 universities were more likely to rate the chance of coordinators reporting the misconduct to be higher (df:6, *p* = 0.012).

### Perception of organisational influence on research misconduct

Among our participants, 78% deemed the investigator competitiveness high or very high in their workplaces. No substantiated difference was observed in pressure on investigators to obtain external funding among various university types (df:6, *p* = 0.054). However, academics in different tenure categories rated this pressure differently (df:9, *p* = 0.008), with the highest difference in the mean scores between TC1 (2.7, SD:0.87) and TC4 (2.4, SD: 0.77). Similarly, a distinction was noted among scholars in various tenure categories in their perception about workplace pressure to obtain tenure (df:9, *p* < 0.001). On average, respondents from TC1 (3.4, SD:0.718) rated this pressure more than the other tenure categories.

Among the participants, 18.5% perceived the effectiveness of their associated organisations’ rules for reducing research misconduct to be high or very high. Lower ratings were more likely to come from subjects in type 1 universities than others (df:6, *p* = 0.0078). Furthermore, people aware of misconduct were more likely to rate their institutional policies’ effectiveness as lower compared to others (df:3, *p* < 0.001). Chances of getting caught were rated as low or very low by 80.6% of respondents. Based on university type or tenure category, no evidence of a difference was found in participants’ ratings of chances of getting caught or severity of penalties in their workplaces. Those who were aware of research misconduct occasions were more likely to rate the chances of getting caught higher than those who did not (df: 3, *p* < 0.001).

### Behavioural influences on research misconduct

**Fig. 2:**
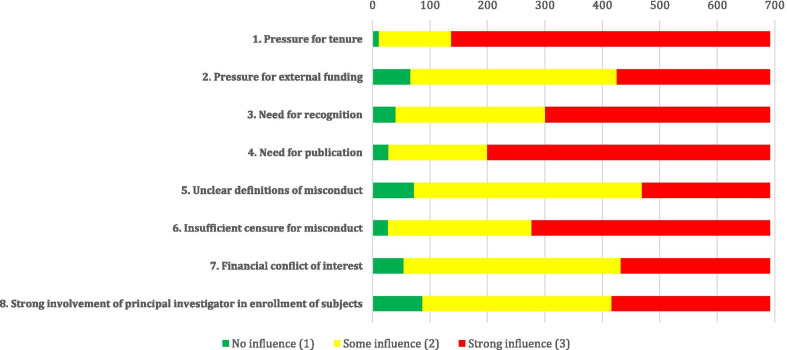
The number of responses to each item in the “behavioural influences on scientific misconduct” section

Pressure for tenure was identified as the most commonly perceived as having a strong behavioural influence on engaging in research misconduct, with a rate of 80.2% among Iranian medical academic scholars. Three other factors were also deemed to have strong behavioural effects on doing misconduct by more than half of our respondents, including the need for publication (71.1%), insufficient censure (punishment) for misconduct occasions (60%), and need for recognition and getting reputed (56.5%). Figure 2 shows the behavioural influences of the scholars on research misconduct.

### Attitudes and beliefs about scientific misconduct

Nearly three-quarters of our respondents (74.4%) were concerned (agreed or completely agreed) about the amount of misconduct in general. Additionally, most of our respondents (91.2%) believed (agreed or completely agreed) that all professional education programs should include information about standards of research ethics. More than four-fifths of those who responded (83.9%) did not believe (disagreed or completely disagreed) that dishonesty and misrepresentation of data are common in society and do not really hurt anybody.

### Publication pressure

The overall cumulative score to all the items in the publication pressure section was 49.5. Nearly 70% of the respondents agreed that their publication output would be of higher quality if there were no publication pressure. Among them, 80.9% suspected that publication pressure leads to data manipulation in some colleagues. The scholars in TC1 were more likely to believe that without publication pressure, their scientific output would have been of higher quality (df:12, *p* = 0.017). Additionally, scholars unaware of misconduct were more likely to find the university’s scientific output criteria for their appointment and reappointment as stimulating (df:4, *p* = 0.001). For more details about the responses’ statistics in each section of the PRMQ, please note Additional file 1, Additional file 2, Additional file 3, Additional file 4, Additional file 5,: Tables 1 to 5.

## Discussion

After devising a reliable and valid questionnaire in Persian (PRMQ), we conducted a national survey in Iran’s medical universities to assess the status quo about scientific misconduct in faculty members. This study is the first interactive survey of medical faculty members (seeking answers from the faculty members and not a mere analysis of their official databases) in Iran about their perceptions of research misconduct on a national scale to the best of our knowledge. Our study tried to reach a random sample of the faculty members, hence validating our survey sample’s representativeness among different university types and scholars in Iran.

However, we are uncertain whether the perception of academic staff about the prevalence of misconduct in this study accurately reflects the true prevalence of misconduct in the participants’ work settings. On the other hand, we indirectly asked the scholars about various instances of misconduct (i.e. plagiarism, data fabrication, etc.), as if they have seen such wrongdoings in their workplaces (and not necessarily conducted by themselves). This could expand the coverage of misconduct identifications as one individual in each department would suffice to report the cases.

A recent survey of plagiarism among various demographics of researchers in Iran reported the percentage of plagiarism at around 30%—based on experts’ opinions [26]. Be that as it may, a survey by Hadji et al. in 2018 [19] directly assessed the prevalence of publication misconduct among corresponding Iranian researchers who had published in Scopus-indexed journals during 2009–2011. Prevalence rates for plagiarism, methodology falsification and data fabrication were reported at 4.9%, 12.65%, and 4.15%, respectively. However, our survey participants claimed to have frequently faced similar misconducts (plagiarism, data falsification, and data fabrication) with a frequency of 12.3%, 20.8%, and 12.4%, sequentially. Both ours and Hadji and her colleagues’ surveys have represented authorship issues as the most common misconduct in Iran. Hadji et al. reported 18.1% of guest authorship in their findings, while we found that 31.2% of medical faculty members deemed to have frequent disagreements about authorship.

Pressure for obtaining tenure (*p* < 0.001) and external funding (*p* < 0.05) was found to be more heeded to by younger medical academic members (TC1) compared with the older scholars (TC4). Early-career scholars were also more likely to believe that without publication pressure, their scientific output would be of higher quality (*p* < 0.05). This is previously addressed by Holtfreter et al. [27], who claimed that professional strains and stressors like publication pressure and pressure to secure external funds are among the most important causative factors of research misconduct. Medical faculty members in Iran are promoted based on the quantity more than the quality of their publications and also are unofficially monitored by their scientometric indices. In the absence of a well-established qualitative promotion model in the current academic system of Iran to assess the faculty members, these professional strains tend to aggravate notably. Furthermore, the respondents perceived the “authorship disagreements” to have the highest number and percentage of “frequently seen” cases among the other misconduct behaviours (*N* =216, 31.2%). This further proves the existing pressure on medical faculty members in Iran to reach higher academic ranks in the least time possible.

The sensitivity to research misconduct cases has significantly risen in academic working environments. Considering the recent endeavours of the National Committee of Ethics in Biomedicine of the Ministry of Health and Medical Education in Iran (including several national guidelines about the status and management of research misconduct in academic medical institutions, capacity building among institutional review boards on national and regional scales to enhance awareness of scientific misconduct, and national decrees about the disciplinary process of regulating the breaching scholars) [10, 28], the rising awareness of medical faculty members in Iran is foreseeable. Our respondents perceived the occurrence of misconducts to be higher as “occasional cases” rather than “frequent cases’ in all of the questions in the perceived prevalence of scientific misconduct subscale. This can imply an indirect source of awareness about the misconducts. Such cases have been echoed more frequently in the workplace due to an increased sensitivity to scientific misconduct. In addition to activities mentioned above, ratification of “the Act for Prevention and Fighting against Fraud in Scientific Publications” by the Iran Parliament in 2017 and its executive bylaw by the Council of Ministers in 2019 could be known as one of the main factors that increased the sensitivity toward scientific misconduct in the academic sphere.

The low perception of institutional policies’ effectiveness against the misconducts in type 1 universities could be due to higher numbers of running research projects and better communication settings for researchers, whereby one perceives the policies not to be sufficient to control the occasions of misconduct [28]. In other words, they are more likely to get aware of misconducts in their workplaces arbitrarily. Predictably, people aware of misconduct were more likely to rate this effectiveness as low (*p* < 0.001) and more likely to rank the chances of getting caught higher than those unaware of such occasions of misconduct (*p* < 0.001). Moreover, participants aware of misconduct were more likely to indicate that a typical research coordinator would probably do nothing when witnessing misconduct by their team members (*p* < 0.001). These are alarming perceptions that need to be effectively addressed before getting entrenched in the early- and mid-career medical researchers in Iran. It is imperative to hold awareness programs in faculty members, aiming to reinforce research ethics and inform them about the probable consequences of such actions. This is in line with the findings of Mardani et al. [13], who reiterated the importance of addressing organisational and managerial monitoring interventions (meso-level activities) to achieving research integrity at the individual level among Iranian medical researchers (micro-level activities).

## Limitations

Our study’s figures should be interpreted with caution since the disposition of the misconduct was not specified in the questionnaire, and not all reported instances may have matched the same definitions. They were also subjective to the respondents’ understanding of each type of fraud [29]. As a limitation, our response rate was only 13.8%. Nevertheless, this figure might be acceptable, considering the busy schedule of medical faculty members in Iran.

Using a qualitative approach to assess the research misconduct in Iranian medical faculty members might have helped gather richer and more comprehensive findings and would be valuable to be conducted in the future. Nevertheless, the source questionnaires were meticulously chosen for translation to best reflect the research misconduct status quo in Iran, and they both used a quantitative approach. Using qualitative items might further decrease the response rate as it could have taken a longer period to answer open-ended questions.

Given that our survey was anonymous, the only intervention to boost the response rate was a second round of emailing to the non-respondents. As some items in the publication pressure section assessed the respondent’s experienced stress and considering the COVID-19-imposed stresses on individuals in general [30], we did not do a third round of emailing during the COVID-19 pandemic. Another limitation might be that the definition of misconduct used in Iran is broader than that used in other countries or other surveys, making it difficult to make comparisons.

## Conclusions

While addressing the limitation of our study, a significant proportion of medical faculty members in Iran had been aware of some research misconduct cases in the past. Granted, there is a viable need to continually educate medical researchers about the repercussions of scientific misconduct, yet more adaptive approaches to mitigate professional strains on early-career researchers are advisable.

## Supplementary Information

**Additional file 1**. The number, mean score and median score of responses to each item in the “perception of workplace environment” section.

**Additional file 2**. The number, mean score and median score of responses to each item in “awareness of scientific misconduct” section.

**Additional file 3**. The number, mean score and median score of responses to each item in the “reporting of scientific misconduct” section.

**Additional file 4**. The number, mean score and median score of responses to each item in the “attitudes and beliefs about scientific misconduct” section.

**Additional file 5**. The number, mean score and median score of responses to each item in the “publication pressure questionnaire” section.

## Data Availability

The datasets generated and analysed during the current study are not publicly available but are available from the corresponding author on reasonable request. The codes applied in this study are publicly available on the GitHub platform (https://github.com/ErfanShamsoddin/ResearchMisconductIran).

## Abbreviations

COVID-19: Coronavirus disease 2019
PRMQ: Persian version of the research misconduct questionnaire
ISID: Iranian Scientometric Information Database
TC: Tenure category
PI: Principal investigator
SD: Standard deviation
