# Supplementary material for: Assessing research misconduct in Iran: a perspective from Iranian medical faculty members
Source: BMC Med Ethics. 2021 Jun 21;22:74. doi: 10.1186/s12910-021-00642-2 (PMC8215315; doi:10.1186/s12910-021-00642-2)
Supplement: Supplementary file 1 — Additional file 1. The number, mean score and median score of responses to each item in the “perception of workplace environment” section. [file 12910_2021_642_MOESM1_ESM.docx]

**Additional Table 1.** Number, mean score and median score of responses to each item in the "perception of workplace environment" section.

| **Item** | **Very low (1)** | **Low (2)** | **High (3)** | **Very high (4)** | **Mean score (S.D.)** | **Median (Q1, Q3)** |
| --- | --- | --- | --- | --- | --- | --- |
| 1. Investigator competitiveness | 35 (5.1%) | 117 (16.9%) | 193 (27.9%) | 347 (50.1%) | 2.7 (0.77) | 3 (2,3) |
| 2. Pressure on investigators to obtain tenure | 13 (1.9%) | 76 (11%) | 308 (44.5%) | 295 (42.6%) | 3.2 (0.73) | 3 (3,4) |
| 3. Pressure on investigators to obtain external funding | 58 (8.4%) | 302 (43.6%) | 222 (32.1%) | 110 (15.9%) | 2.5 (0.85) | 2 (2,3) |
| 4. Severity of penalties for scientific misconduct | 215 (31.1%) | 356 (51.4%) | 100 (14.5%) | 21 (3%) | 1.8 (0.75) | 2 (1,2) |
| 5. Chances of getting caught for scientific misconduct if it occurs | 164 (23.7%) | 394 (56.9%) | 123 (17.8%) | 11 (1.6%) | 1.9 (0.69) | 2 (2,2) |
| 6. Investigators’ understanding of rules and procedures related to scientific misconduct | 75 (10.8%) | 406 (58.7%) | 202 (29.2%) | 9 (1.3%) | 2.2 (0.63) | 2 (2,3) |
| 7. My own understanding of rules and procedures related to scientific misconduct | 9 (1.3%) | 175 (25.3%) | 419 (60.5%) | 89 (12.9%) | 2.8 (0.64) | 3 (2,3) |
| **Additional Table 1 (Continued).** | | | | | | |
| **Item** | **Very low (1)** | **Low (2)** | **High (3)** | **Very high (4)** | **Mean score (S.D.)** | **Median (Q1, Q3)** |
| 8. Other research staff's understanding of rules and procedures related to scientific misconduct | 4.3 (6.2%) | 362 (52.3%) | 272 (39.3%) | 15 (2.2%) | 2.3 (0.63) | 2 (2,3) |
| 9. Investigators’ support of rules and procedures related to scientific misconduct | 73 (10.5%) | 450 (65%) | 156 (22.5%) | 13 (1.9%) | 2.1 (0.61) | 2 (2,2) |
| 10. Research coordinators’ support of rules and procedures related to scientific misconduct | 116 (16.8%) | 368 (53.2%) | 185 (26.7%) | 23 (3.3%) | 2.1 (0.73) | 2 (2,3) |
| 11. Other research staff’s support of rules and procedures related to scientific misconduct | 111 (16%) | 423 (61.1%) | 141 (20.4%) | 17 (2.5%) | 2.0 (0.67) | 2 (2,2) |
| 12. The effectiveness of your organization’s rules and procedures for reducing scientific misconduct | 179 (25.9%) | 385 (55.6%) | 116 (16.8%) | 12 (1.7%) | 1.9 (0.70) | 2 (1,2) |

SD: Standard deviation; Q1: The first quartile; Q3: The third quartile.
