# Supplementary material for: Assessing research misconduct in Iran: a perspective from Iranian medical faculty members
Source: BMC Med Ethics. 2021 Jun 21;22:74. doi: 10.1186/s12910-021-00642-2 (PMC8215315; doi:10.1186/s12910-021-00642-2)
Supplement: Supplementary file 2 — Additional file 2. The number, mean score and median score of responses to each item in “awareness of scientific misconduct” section. [file 12910_2021_642_MOESM2_ESM.docx]

**Additional Table 2.** Number, mean score and median score of responses to each item in “awareness of scientific misconduct” section.

| **Item** | **Never** | **Once** | **2-5times** | **6-10 times** | **>10 times** | **Mean score (SD)** |
| --- | --- | --- | --- | --- | --- | --- |
| 1. In your work environment, how often have you been aware that an investigator engaged in scientific misconduct during the past year? | 175 (25.3%) | 151 (21.8%) | 271 (39.2%) | 61 (8.8%) | 34 (4.9%) | 2.4 (1.1) |
| 2. In your work environment, how often have you been aware that research coordinator or other personnel engaged in scientific misconduct during the past year? | 349 (50.4%) | 120 (17.3%) | 148 (21.4%) | 38 (5.5%) | 37 (5.3%) | 1.9 (1.1) |
| **Item** | **Observed myself or heard from colleagues** | **Through official channels of my organisation** | | | | **Mean score (SD)** |
| 3. How did you learn about the instances of scientific misconduct you are aware of | 499 (72.1%) | 193 (27.9%) | | | | NA |

SD: Standard deviation; Q1: The first quartile; Q3: The third quartile.
