# Supplementary material for: Assessing research misconduct in Iran: a perspective from Iranian medical faculty members
Source: BMC Med Ethics. 2021 Jun 21;22:74. doi: 10.1186/s12910-021-00642-2 (PMC8215315; doi:10.1186/s12910-021-00642-2)
Supplement: Supplementary file 3 — Additional file 3. The number, mean score and median score of responses to each item in the “reporting of scientific misconduct” section. [file 12910_2021_642_MOESM3_ESM.docx]

**Additional Table 3.** Number, mean score and median score of responses to each item in the "reporting of scientific misconduct" section.

| **Item** | **Probably nothing** | **Oppose but not report** | **Ask to report, otherwise report themselves** | **Will report the misconduct** | **Mean score (S.D.)** | **Median (Q1, Q3)** |
| --- | --- | --- | --- | --- | --- | --- |
| 1. What do you think a typical research coordinator in your area would do if they knew a principal or co-investigator violated accepted rules for research integrity on a project or assignment? | 237 (34.2%) | 268 (38.7%) | 68 (9.8%) | 119 (17.3%) | 2.0 (1.0) | 2 (1,3) |
| 2. What do you think a typical research coordinator in your area would do if they knew a member of a research team or staff member violated accepted rules for research integrity on a project or assignment? | 225 (32.5%) | 257 (37.1%) | 48 (6.9%) | 162 (23.4%) | 2.2 (1.1) | 2 (1,3) |
|  | Not at all likely | Somewhat likely | Very likely | | Mean (S.D.) | Median (Q1, Q3) |
| 3. If someone engaged in scientific misconduct and was reported to your institutional authorities, how likely do you think it is that they would be disciplined? | 216 (31.2%) | 409 (59.1%) | 67 (9.7%) | | 1.7 (0.60) | 2 (1,2) |

SD: Standard deviation; Q1: The first quartile; Q3: The third quartile.
