# Supplementary material for: Assessing research misconduct in Iran: a perspective from Iranian medical faculty members
Source: BMC Med Ethics. 2021 Jun 21;22:74. doi: 10.1186/s12910-021-00642-2 (PMC8215315; doi:10.1186/s12910-021-00642-2)
Supplement: Supplementary file 4 — Additional file 4. The number, mean score and median score of responses to each item in the “attitudes and beliefs about scientific misconduct” section. [file 12910_2021_642_MOESM4_ESM.docx]

**Additional Table 4.** Number, mean score and median score of responses to each item in the "attitudes and beliefs about scientific misconduct" section**.**

| **Item** | **Completely disagree (1)** | **Disagree (2)** | **Indecisive (3)** | **Agree (4)** | **Completely agree (5)** | **Mean score (S.D.)** | **Median (Q1, Q3)** |
| --- | --- | --- | --- | --- | --- | --- | --- |
| 1. I am concerned about the amount of misconduct | 8 (1.2%) | 44 (6.4%) | 125 (18.1%) | 344 (49.7%) | 171 (24.7%) | 3.9 (0.8) | 4 (3,4) |
| 2. I think the responsibility for the scientific integrity of a study lies with the principal investigator only | 118 (17.1%) | 340 (49.1%) | 43 (6.2%) | 149 (21.5%) | 42 (6.1%) | 2.5 (1.1) | 2 (2,4) |
| 3. All professional education programs should include information about standards of research ethics | 6 (0.9%) | 13 (1.9%) | 42 (6.1%) | 354 (51.2%) | 277 (40%) | 4.2 (0.7) | 4 (4,5) |
| 4. I feel uncomfortable talking with RCs and other research personnel about their ethical behavior | 46 (6.6%) | 180 (26%) | 157 (22.7%) | 234 (33.8%) | 75 (10.8%) | 3.1 (1.1) | 3 (2,4) |
| 5. Dishonesty and misrepresentation of data is common in society and doesn’t really hurt anybody | 345 (49.9%) | 235 (34%) | 58 (8.4%) | 40 (5.8%) | 14 (2%) | 1.7 (0.9) | 2 (1,2) |

SD: Standard deviation; Q1: The first quartile; Q3: The third quartile.
